# LitMiner: integration of library services within a bio-informatics application

**DOI:** 10.1186/1742-5581-3-11

**Published:** 2006-10-19

**Authors:** Jeffrey Demaine, Joel Martin, Lynn Wei, Berry de Bruijn

**Affiliations:** 1 CISTI Research, Canada Institute for Scientific and Technical Information, National Research Council of Canada, 1200 Montreal rd. Ottawa, Ontario, K1A 0R6, Canada; 2 Interactive Information, Institute for Information Technology, National Research Council of Canada, 1200 Montreal rd. Ottawa, Ontario, K1A 0R6, Canada

## Abstract

**Background:**

This paper examines how the adoption of a subject-specific library service has changed the way in which its users interact with a digital library. The LitMiner text-analysis application was developed to enable biologists to explore gene relationships in the published literature. The application features a suite of interfaces that enable users to search PubMed as well as local databases, to view document abstracts, to filter terms, to select gene name aliases, and to visualize the co-occurrences of genes in the literature. At each of these stages, LitMiner offers the functionality of a digital library. Documents that are accessible online are identified by an icon. Users can also order documents from their institution's library collection from within the application. In so doing, LitMiner aims to integrate digital library services into the research process of its users.

**Methods:**

Case study

**Results:**

This integration of digital library services into the research process of biologists results in increased access to the published literature.

**Conclusion:**

In order to make better use of their collections, digital libraries should customize their services to suit the research needs of their patrons.

## Background

### Introduction

LitMiner is a work-centered digital information application that incorporates aspects of text analysis, visualization, and digital library services. This paper will focus on the use of LitMiner as an extension of the library's services. The various techniques in LitMiner for manipulating gene information will be discussed in subsequent articles.

**Figure 1:**
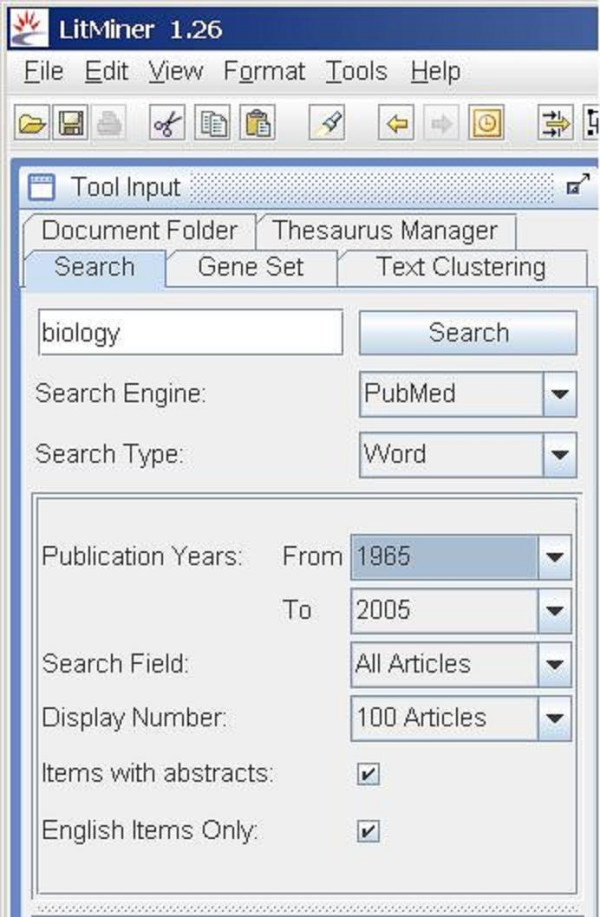
**Tool tabs**. The functionality of LitMiner is grouped under five tabs. The most basic, Search, is highlighted.

The LitMiner project [1-4] is a collaborative effort in bioinformatics between three institutes within the National Research Council of Canada (NRC). The Institute for Information Technology (IIT) conceived LitMiner with the goal of enabling biologists from the Institute for Biological Sciences (IBS) to analyze the text of published articles. Access to the published literature of biology is provided by the library of the National Research Council (the Canada Institute for Scientific and Technical Information, or "CISTI"), through either its document delivery services or via its electronic collection. This bioinformatics tool helps the biologists in identifying genes and in exploring the relationships between genes as suggested by the literature. The functionality of LitMiner is grouped into five tabs (see Figure 1).

### Customization of digital library access

Over the past decade, libraries have adopted web technologies to better meet the needs of their patrons. From simple web pages to fully functional digital libraries, users have gained easier access to the information they seek, while libraries have remained relevant to users by developing increasingly sophisticated information services.

The advent of electronic publishing has made the library's collection accessible from the user's desktop. An early example of this is the DADS system ("DTV Article Database System") [5], which laid out some of the principles that an "electronic article database service" should follow: the integration of data from disparate sources into a common user interface, as well as the integration of document ordering and delivery mechanisms.

Moving beyond this basic provision of access to electronic resources, current thinking about the design of digital libraries emphasizes the customization of access and its integration within the intellectual workflow of the researcher: Soergal [6] writes that a guiding principle of digital library design is that "Information access must be embedded seamlessly into an integrated system that supports all of a user's work...".

Rather than a generic interface for all, customized views of the collection permit a new type of relationship between the library and its patrons. One way in which a library can fine tune how users access its collection is through services that target the information needs of a given user group, in what Clifford Lynch has described as "customization by community" [7]. The various instances of the MyLibrary project enhance the quality of access to their digital libraries by developing specialized services that are focused on specific user groups [8].

The customization of LitMiner to the needs of a group of biologists is in keeping with current trends in the library community [see [9]]. While LitMiner may not be the fastest application in any one respect, it is unique in the range of functionality that it offers. From clustering to gene-analysis, and from visualization to document ordering, LitMiner allows users to quickly shift from one method of exploring gene relationships to another. For the library, the integration of digital library services into a subject-specific text analysis application represents a value-added service that differentiates the library from other sources of information such as search engines.

### Integration into the workspace

As with other work-centered information services such as DAFFODIL, MatDL, and MyLibrary (described below), the usefulness of LitMiner is a function of the seamlessness with which users can access the collection. The design of LitMiner is a major step in this direction, as it offers a suite of tools that enables users to focus on interacting with information rather than switching between applications. By handling the mechanics of searching and retrieving articles from the collection, users are able to quickly access articles as part of the data-mining process. LitMiner offers automatic authentication, one-button ordering, and identification of accessible online articles, within both the library's collection as well as those external articles that are freely available.

Although LitMiner uses one of the library's pre-existing ordering mechanisms to handle orders, the involvement of the library in the development of this application is a departure from previous product development initiatives of the library. Rather than designing a stand-alone library service, this approach works with software development in other institutes such that the library is integral to its design, and not an afterthought.

### Related research

Several projects have also focused on customizing digital libraries for the needs of users. Each provides an example of the different ways in which the standard search-and-retrieval functions of a digital library can be enhanced.

▪ The DAFFODIL application offers search stratagems for designing complex queries of federated databases [10]. In addition, DAFFODIL visualizes the results of certain queries using a network graph.

▪ The MatDL project is a repository of materials science data. This web-based application goes beyond the basic search functionality of a digital library in that it enables submissions to the repository from the researchers' workspace [11].

▪ The MyLibrary project provides a user-customizable portal to the digital library [8]. It enables users to manage their preferences, helping the library to target its services [12,13].

▪ The MIRRORS system implements multiple agents to perform automated medical information retrieval. The system achieves high-precision results by basing its searches on patient records and known ("trusted") sites. Results are associated with a patient's file such that the information is integrated into the physicians' workflow [14].

In addition, several commercial applications are available that provide users of PubMed with an integrated search environment. While stand-alone applications such as Skolar MD [15] and Quosa [16] provide different functionality than LitMiner, they also illustrate how PubMed searching can be integrated into the workflow of the user.

Skolar MD is an example of digital library content integrated into a work-centered information tool. Designed for use by physicians in the context of their clinical practice, Skolar MD provides access to a range of biomedical content besides PubMed, including Ovid databases. A product of a scientific publishing house, Skolar MD exemplifies the need for digital library services that are designed to be used in situ, and that provide access to specialized information that can be put into practice immediately.

Quosa is designed to help researchers search PubMed and other databases, and organize their personal collections of articles. It offers more document-management features than does LitMiner, but none of the gene-analysis techniques (such as a visualization of the co-occurrence of genes in the literature). Unlike Skolar MD, Quosa is not associated with any licensed content and was developed by an independent company. That is, without subscriptions to the electronic content of scientific publishers, Quosa will only be able to retrieve articles that are free. In that sense, Quosa is most practical for users of an institution that has paid the licensing fees to access the literature, such as a library. It would be helpful to creators of digital libraries if Quosa could also be integrated into an institution's digital library service. That is, if Quosa had an application programming interface (API), a library could design its own software around this search tool. This modularization of searching and content would provide libraries with great flexibility in designing information tools targeted at specific user groups.

A feature that these tools offer that could enhance LitMiner is the ability to export search results in a standard format readable by citation manager software such as ProCite or EndNote. This feature would make LitMiner broadly compatible with other applications in use by academics, further integrating itself into the work process of the biologists.

These projects are all examples of what Wilensky [17] describes as work-centered information services. These are digital library applications that are adapted to the needs of a particular user-group. According to Wilensky, the characteristics of these users are that they:

▪ Are more interested in interacting with information than in simply retrieving documents.

▪ Want to build and access their own internal collections.

▪ Want an information system that will integrate into their work practices. This may require a customized interface.

The digital library is often organized along the lines of the information architecture, such as separate search interfaces for the catalogue and for indexing-service databases (i.e.: SilverPlatter). The distinctions between these types of searches are irrelevant to users, who seek an answer, regardless of which bin it may be found in.

In contrast, another way of organizing the digital collection that is arguably more convenient for the user is to package the access as part of a more focused service (as exemplified by the applications discussed above). Here the emphasis is not on the comprehensiveness of the collection, but on the seamlessness of access. Instead of a one-size-fits-all digital library, a customized service provides easier interaction with a subset of the digital library. As an example of a work-centered information service, LitMiner emphasizes the integration of existing library technologies into a customized digital information service.

## Methods

### Implementation

The LitMiner application provides users with a suite of tools for searching the biomedical literature and for manipulating the results. Figure 1 shows the left-hand frame of the interface. The functionality of LitMiner is grouped into five sections:

• The *Search *tab (highlighted in Figure 1) links to PubMed and is the main method of bringing textual information into LitMiner for manipulation.

• The *Text Clustering *tool clusters the results into a hierarchical subject list based on keywords extracted from the titles and abstracts of the articles.

• The *Document Folder *enables users to save the results of their searches locally and to share these document sets with other users.

• The *Gene Set *enables users to compare the co-occurrence of genes in the literature, and to visualize the relationships between genes using a network graph.

• The *Thesaurus Manager *allows users to fine-tune the aliases that are used to refer to genes in searching.

**Figure 2:**
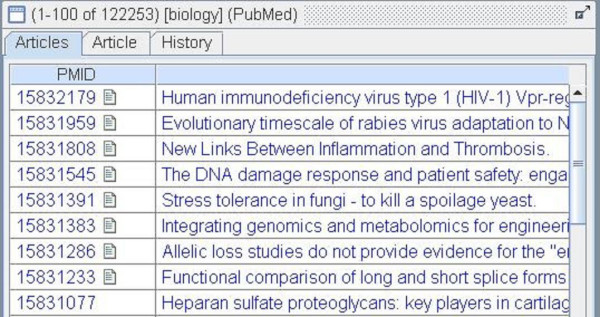
**Articles table**. The results of the search.

The LitMiner application is written entirely in Java. Although not web-based, the architecture follows a client-server model. While the client performs much of the processing, all interactions with the databases are routed through the server. Note that this paper does not discuss the gene-alias matching which is one of the facets of the LitMiner application. For a discussion of this technique, the reader is referred to [1,2,4]. This paper will focus on the provision of access to articles in LitMiner and how the immediacy of this access within LitMiner has changed how users interact with the digital library. While each of the five tools in the application serves a separate purpose, they each provide an "articles" panel (see Figure 2). This enables users to retrieve articles as part of their manipulation and filtering of information (for example interacting with a graph or clustering documents). Rather than making the digital library a distinct entity within LitMiner access that the digital library provides is integrated into each facet of the application.

Records retrieved by a search are displayed in a table that lists article titles. Those records that are accessible online have a small icon associated with them (see Figure 2). A list of articles which are available online was compiled separately and the URL of each, along with an identifier of the article's location, was recorded in a database. Three ways of identifying accessible articles were used:

1. The PubMed record contains a DOI (digital object identifier) in the MEDLINE "AID" field. When the user clicks on the "accessible online" icon, the LitMiner application launches a web browser and loads a website that resolves DOIs [18]. If successfully resolved, the document (typically in pdf format) is displayed.

2. The article is held in PubMed Central, and is likely an OpenAccess article. It is therefore freely accessible. When the user clicks on the "accessible online" icon, the LitMiner application launches a web browser and loads PubMed's article resolver [19] with the article's PMID number as the final argument in the http call.

3. The PubMed record is associated with a journal to which CISTI is subscribed. An OpenURL link resolver was used to translate the bibliographic data of an article into a URL (typically of the publisher's website) that gives access to the PDF file of that article.

In this way, the application is able to provide access to articles from many different sources (be it a publisher's server, PubMed Central, or a scholarly society's repository of articles) in an integrated way. The resulting access to the desired document is seamless for the user, whether it is a subscription-only article available by virtue of CISTI's licences with various publishers or an article freely available on the Web. In effect, the result of pre-compiling the location of articles known to be accessible is to extend the breadth of the collection.

From the list of results, double-clicking on any given row will cause the bibliographic details of that article to be displayed in another panel. From this article display panel several options are available to users. If the user chooses to view an online document, a Web browser is automatically launched and the associated file (typically a PDF document located on PubMed Central or on a publisher's Web site) is displayed.

**Figure 3:**
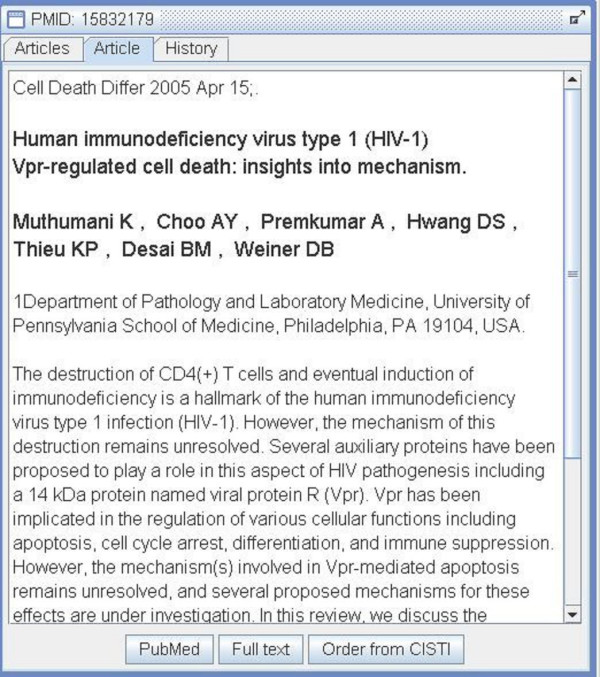
**Article detail display**. The abstract of an article is shown in the article detail display. Note the buttons at the bottom of the display allowing the user to view the full text of the article, to view the record in PubMed, or, if those links fail, to order the article from CISTI.

Those documents which are not identified as being accessible online can be ordered via CISTI's document delivery service. A button in the article detail display (see Figure 3) sends the bibliographic information for the request to CISTI's Blank Web Order form. This feature is convenient for the user, as they are not required to open a Web browser, navigate to the appropriate order form within CISTI's digital library, and manually type the bibliographic information relating to the article of interest into the Blank Web order form.

At CISTI the physical journal is located and scanned. The (now electronic) document is then delivered to the user. If CISTI has the journal on hand, the time required to fill such an order can be very short, often the same day. This makes the backfiles of print subscriptions as accessible as the most recent, born-digital articles.

Each order, whether online or through CISTI, is logged to the LitMiner server. This enables an analysis of user ordering patterns. At a high level, the overall number of orders indicates the utility of the service. More detailed study of the journals, authors and subjects most frequently ordered could lead to improved library services.

### Example

Between February 2004 and February 2005, three biologists who belong to the core user group and who volunteered their ordering data ordered 360 documents through LitMiner (see Table 1). Of these, 246 (68%) were electronic documents which were freely available online (i.e.: OpenAccess journals, articles in PubMed Central, and articles with a Document Object Identifier in their MEDLINE record). In addition, 114 (32%) articles were ordered from CISTI's collection via LitMiner.

**Table 1:** The number of documents ordered by three biologists. The orders are broken down by year and by ordering method.

**User**	**Application used for accessing/ordering**	**Order type**	**February 1 → January 31**			
			**2001**	**2002**	**2003**	**2004**
**1**	Virtual Library	Blank order form	35	46	65	7
		Loansome Doc	0	0	0	0
	LitMiner	Order from CISTI	0	0	0	65
		Online article	n/a	n/a	n/a	58
**2**	Virtual Library	Blank order form	0	0	0	0
		Loansome Doc	86	75	91	104
	LitMiner	Order from CISTI	0	0	0	4
		Online article	n/a	n/a	n/a	14
**3**	Virtual Library	Blank order form	52	47	13	0
		Loansome Doc	0	0	105	83
	LitMiner	Order from CISTI	0	0	0	48
		Online article	n/a	n/a	n/a	324

In registering for Loansome Doc ordering, the user has notified PubMed that CISTI is their library. Upon locating an article of interest in PubMed, a user can easily order it from CISTI by sending the article's bibliographic information to the Loansome Doc service. In this way, Loansome Doc is a customized ordering service that is integrated into the PubMed search interface.

## Results

The data on the number of articles that users have ordered from CISTI goes back to 2001 (see Table #1). As a result it is possible to see the effect that LitMiner has had on ordering patterns of its users since it became available to users in early 2004.

Over the period 2001 to 2003, the number of orders that user #1 placed using the Virtual Library's Blank Order form nearly doubled from 2001 to 2003. That user began using LitMiner in 2004 and this new application became the ordering method of choice for 95% of the user's orders. Usage of the Blank order form has fallen to a fraction of its former usage. In the context of work, this biologist now uses LitMiner as the primary method of accessing the library's collection and for ordering documents.

Over the same three years, user #2 never used the Virtual Library's Blank order form. Instead, this user has been a heavy user of Loansome Doc, as it is a convenient way to access the literature. The number of articles ordered or accessed continued to grow in 2004, with LitMiner accounting for some 15% of these. The use of LitMiner has not significantly changed how this user orders documents.

User #3 has been a steady user of the Virtual Library's Blank Order form. In 2003, this user began ordering some documents via Loansome Doc. The following year the LitMiner application was introduced and this user altogether stopped using the Blank Order form, showing a preference instead for Loansome Doc and a very large number of online order through LitMiner.

## Discussion

The data shows how three users, each with a different way of interacting with the library, have modified their habits as a result of using LitMiner. For two users (#1 and #3) LitMiner has become a heavily used method for ordering documents from the library. The seamlessness with which documents can be ordered from the digital library via LitMiner has resulted in users ceasing to use the Blank Order form or (in the case of user #2) ordering concurrently with Loansome Doc.

User #2's habit of ordering from Loansome Doc is instructive. Loansome Doc is a service of the National Library of Medicine that acts as a mediator between the PubMed database and the user's library. By registering with Loansome Doc, a user can order articles in their library's collection from within PubMed. Conceptually, the integration of Loansome Doc ordering within PubMed is similar to the incorporation of CISTI ordering within LitMiner. In both cases the application manages the user's library account information. The result is transparent interaction with the digital library through a search interface that compliments rather than encumbers the biologists' information-seeking behaviour.

The number of articles accessed by user #3 is so great that one wonders if it may be too easy to access electronic articles. Whatever the researcher does with 455 articles is beyond the scope of this paper, yet it is clear that the digital library is providing the content that biologist seeks.

As a caveat, it must be noted that it is difficult to isolate how much the integration of the document-ordering mechanism has contributed to the success of LitMiner. While the statistics indicate an increase in the number of documents ordered there is no way to isolate whether the increase was entirely due to the use of LitMiner. That ordering a document by pressing a button is more convenient than manually entering bibliographic information is self-evident. Yet the number of orders recorded could be independent of the use of LitMiner: perhaps users would have ordered an equal number of documents using existing methods anyway.

In addition, we have no way to capture users' accessing of online articles because of a Web search external to LitMiner. This unmeasured downloading of full-text scientific articles is an environmental variable that is orthogonal to the question at hand, as users will undoubtedly continue to find documents by simply browsing the Web regardless of what the library does. The comparison here is between access to digital library services as part of the larger library Web presence and access to digital library services as integrated into a subject-specific tool.

### Further work

Wilensky states that work-centered information services require "improved protocols for client program interaction with repositories". This is certainly the case with LitMiner, where reliable identification of the library's holdings within PubMed results has proven difficult. We are investigating ways in which the OpenURL protocol can be used to identify documents in the library's collection to external applications such as LitMiner based on a Web-services architecture.

The small number of users in this project precludes any meaningful statistical analysis of the data. However, with new users signing up for LitMiner accounts and more data being collected, it is hoped that an adequately powered statistical analysis will be possible in the future.

With the recent addition of a search engine to the LitMiner server, it becomes possible to search local databases of information. This opens up the possibility that alternate versions of LitMiner could be developed for other areas of research.

## Conclusion

LitMiner integrates the functionality of a digital library into a bioinformatics application, bringing the services within easy reach of researchers. Users benefit by being able to access documents of interest from within an application that helps them mine the article abstracts for gene relationships.

Insofar as the library's role is to serve the information retrieval needs of its clients, the data shows that a work-centered information service such as LitMiner can facilitate user access to information. Preliminary data suggests that the LitMiner system increases user ordering of documents by facilitating access to the literature. It is hoped that by providing a convenient method of interacting with the collection, services that were previously only offered within the digital library can be integrated into the workflow of the biologists. While the full resources of the library are lacking from such a search tool, the advantages of simplified searching, ordering, as well as workflow integration make for a very useful application. These preliminary results suggest that digital library services should be packaged into subject-specific applications.

This paper has shown how a digital library can refine the services it offers allowing users to interact seamlessly with the collection. It shows how users prefer to access a digital library via an interface (whether as a stand-alone application like LitMiner, or integrated into PubMed as is Loansome Doc) that is integrated into their information seeking tasks and that offers more convenient document ordering. Whether the user prefers to order using LitMiner or Loansome Doc, the seamlessness of the interaction is the common element. In order to support work-centered applications, a digital library should:

▪ Identify a user group for which work-centered information services are appropriate (see discussion of Wilensky's criteria, above).

▪ Provide a programmatic interface to its collection.

▪ Work with developers to make access to its collection integral to the tools that the users employ.

▪ Record users' orders and access of digital publications from the work-centered application so as to inform collection development and quantify usage.

In an environment where ease of access to the full text of scientific articles is taken for granted, libraries would do well to pursue technologies that enable access to their resources to be incorporated into the user's desktop environment. A digital library can be presented differently to different users and can be accessible through multiple interfaces. To be more relevant and convenient for users, digital library services should be integrated into work-centered applications.

## Competing interests

The author(s) declare that they have no competing interests.

## Authors' contributions

JM lead the project and developed the text-clustering tool. LW was the main developer, and contributed significant interface design improvements. BDB compiled the databases and developed gene-alias disambiguation algorithms. JD developed searching and document ordering functionality.
